# Age dependence of pulmonary artery blood flow measured by 4D flow cardiovascular magnetic resonance: results of a population-based study

**DOI:** 10.1186/s12968-016-0252-3

**Published:** 2016-05-31

**Authors:** Thomas Wehrum, Paul Hagenlocher, Thomas Lodemann, Werner Vach, Iulius Dragonu, Anja Hennemuth, Constantin von zur Mühlen, Judith Stuplich, Ba Thanh Truc Ngo, Andreas Harloff

**Affiliations:** Department of Neurology, University Medical Center Freiburg, Breisacher Straße 64, 79106 Freiburg, Germany; Institute for Medical Biometry and Statistics, University of Freiburg, Freiburg, Germany; Department of Diagnostic Radiology – Medical Physics, University Medical Center Freiburg, Freiburg, Germany; Fraunhofer MEVIS, Bremen, Germany; Department of Cardiology, University Heart Center Freiburg, Freiburg, Germany

**Keywords:** Population, 4D flow CMR, Pulmonary arteries, Pulmonary blood flow

## Abstract

**Background:**

It was our aim to systematically analyze pulmonary artery blood flow within different age-groups in the general population using 4D flow cardiovascular magnetic resonance (CMR) in order to provide a context for interpreting results of future studies (e.g., in pulmonary hypertension) using this technique.

**Methods:**

An age-stratified sample (*n* = 126) of the population of the city of Freiburg, Germany, underwent ECG-triggered and navigator-gated 4D flow CMR at 3 T of the pulmonary arteries and the thoracic aorta. Analysis planes were placed in the main, left, and right pulmonary artery using dedicated software. Study participants were divided into three groups (1:20–39; 2:40–59; and 3:60–80 years of age). Subsequently, pulmonary blood flow was visualized, quantified and compared between groups.

**Results:**

Time-to-peak of systolic antegrade flow was shorter, peak and average velocities and flow volumes were lower in older subjects. At the end of systole, retrograde flow in the main pulmonary artery was observed in all but one subject. Subsequently, a second antegrade flow peak occurred in diastole which was lower in older subjects. Age was an independent predictor of hemodynamic change after adjustment for cardiovascular risk factors and body-mass-index. During systole, abnormal vortices occurred in the main pulmonary artery in four male subjects.

**Conclusions:**

Comprehensive analysis of pulmonary blood flow was feasible in all subjects. We were able to detect an independent effect of ageing on pulmonary hemodynamics reflecting increased vessel stiffness and reduced pulmonary circulation. Findings of this study may be helpful for discriminating physiological from pathological flow in patients with pulmonary diseases in the future.

**Electronic supplementary material:**

The online version of this article (doi:10.1186/s12968-016-0252-3) contains supplementary material, which is available to authorized users.

## Background

Pulmonary artery blood flow is strongly dependent on adequate cardiac function and absence of systemic vascular disease and pathological conditions of the lungs. Hence, many pathologic conditions may lead to altered pulmonary hemodynamics and elevation of pulmonary arterial pressure [1]. Pulmonary hypertension (PH, i.e., the elevation of mean pulmonary arterial pressure ≥25 mmHg [2]) is a serious condition which requires accurate assessment of intrapulmonary hemodynamics and pressure to identify patients, who will benefit from treatment. Currently, the diagnostic gold-standard for intra-pulmonary pressure evaluation is right-heart catheterization, which is an invasive procedure. Hence, transthoracic echocardiography (TTE) is used as a non-invasive screening tool by assessing cardiac function and estimating pulmonary arterial pressure non-invasively [3]. Unfortunately, specificity of TTE for the identification of patients with PH is limited and [3], therefore, other non-invasive diagnostic tools are under investigation. One promising technique is 4D flow cardiovascular magnetic resonance (CMR), which allows time-resolved and three-dimensional assessment of hemodynamic parameters in vivo [4] and has already been applied to the pulmonary arteries: PH was reported to be associated with a decrease of flow parameters such as peak systolic velocity, peak flow, stroke volume [5], the increase of pulmonary artery (PA) diameter [6] and stiffness [7], early onset of retrograde flow in the main pulmonary artery (MPA) [8], and the occurrence of abnormal vortices [9, 10]. These findings were made by examining PH patients and healthy volunteers [5, 7–9]. Normal pulmonary flow characteristics have previously been described using 2D phase-contrast CMR and echocardiography and have been compared with right heart catheterization indicating a higher agreement of CMR than of echocardiography regarding pulmonary flow and pressure estimation [11]. However, previous studies were limited in sample size [5, 7–9, 11]. Furthermore, 2D flow CMR is of limited use in a clinical setting because it does not allow retrospective selection of regions of interest inside a 3D data volume to perform post-hoc quantification of blood flow parameters. Using 4D flow CMR, all measurements are performed within one dataset with no need for several 2D acquisitions with inherent differences in heartrate [12]. These advantages may allow a more comprehensive study of hemodynamic change during the course of PH. However, reference values derived from the general population are needed to interpret results of future interventional studies.

## Methods

### Study population

We performed a cross-sectional observational study of the population of the city of Freiburg, Germany. Our cohort was established on the basis of data obtained from the local residents’ registration office. We included at least 20 subjects per decade (10 females and 10 males) within the age of 20–80 years. Starting in October 2012, 3500 age-stratified and randomly selected residents of Freiburg were contacted by mail, asked to participate in our study, and provided with details on how to contact the study team. 308 subjects responded to our mail and were contacted by phone on the basis of first-come, first-served. 147 had to be excluded because of reporting CMR contraindications during the telephone interview, too many participants in this group of age, or because no suitable date could be realized. 161 subjects were finally scheduled for study CMR. In 23 subjects the CMR protocol was not completed for technical reasons, 11 did not appear on the appointed day, 11 aborted CMR examination early because of claustrophobia, and 5 were not suited for CMR due to contraindications that were not evident at the preceding telephone interview. Because of insufficient response within the group of 20–29 year old males, the study was advertised on the University Hospital Freiburg intranet and men within this age-interval (*n* = 922) were invited to participate. The first 16 subjects who contacted the study team by email were consecutively included. One person had to be excluded for technical difficulties during CMR and one subject did not arrive on the appointed day. Finally, datasets of 126 subjects were available for analysis.

Cardiovascular risk factors and demographics were determined by interview on site and blood pressure was measured at the left upper arm in a supine position after 5 min rest before and after CMR examination. Heart rate was documented every 5 min during CMR blood flow measurements. All participants underwent additional transthoracic echocardiography (TTE) using a Toshiba Artida system (4.8-2 MHz PST-30BT transducer; Toshiba Medical Systems Corporation, Tokyo, Japan) based on the recommendations and standards of the American Society of Echocardiography [13] on the same day (=median) as CMR. The study was approved by the ethics committee of the University of Freiburg and written informed consent was obtained from all participants.

### 4D flow CMR

All CMR examinations were conducted on a routine 3-Tesla MR system (TIM Trio, Siemens Healthcare AG, Erlangen, Germany), using a standard 12-element body coil. 4D flow CMR was used to obtain time-resolved and three-dimensional blood flow parameters of the pulmonary arteries and the thoracic aorta. All experiments used prospective ECG-gating and navigator-gating to allow free breathing [14]. Parameters of 4D flow CMR were: echo time/repetition time (TE/TR) =2.6/5.1 ms, flip angle =7°, temporal resolution =20.4 ms, matrix size =320 × 240 × 58, bandwidth =450Hz/pixel, spatial resolution =2.1 × 2.1 × 2.5 mm, velocity sensitivity along all three directions =150 cm/s, and parallel imaging (PEAK-GRAPPA) along the phase encoding direction (y) with an acceleration factor of *R* = 5 (20 reference lines).

### Data analysis

**Fig. 1:**
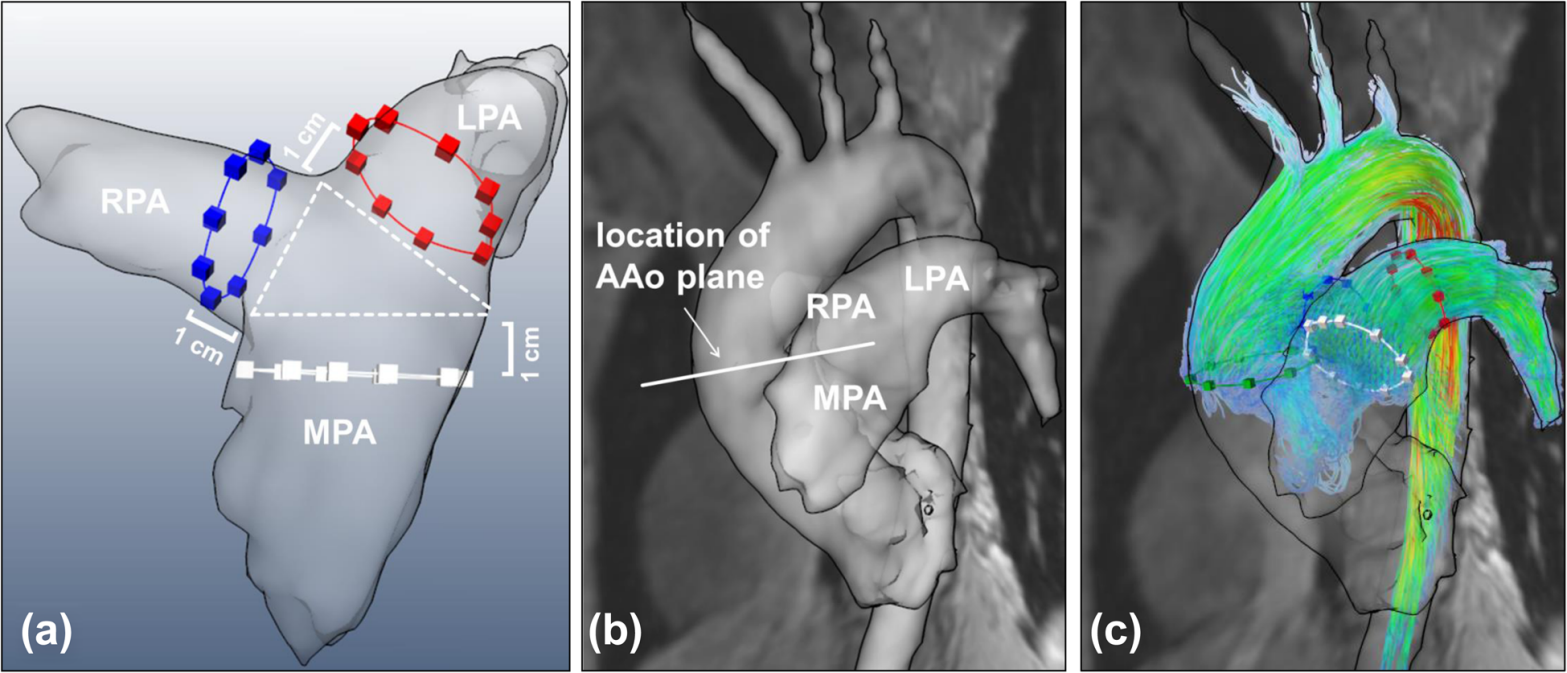
Positioning of analysis planes. **a** Analysis planes in the MPA, LPA, and RPA were positioned with a distance of 1 cm distal to the MPA bifurcation. **b** The AAo plane was located in a standardized fashion on a level with the lower edge of the RPA. **c** 4D flow quantification was performed in all four analysis planes

4D flow CMR datasets were analyzed using MEVISFlow software (Fraunhofer MEVIS, Bremen, Germany) [15]. After corrections for eddy-currents and phase-wraps, the vessel was segmented and three analysis planes were manually positioned perpendicular to the vessel lumen in the main pulmonary artery (MPA), the left pulmonary artery (LPA), and the right pulmonary artery (RPA). All planes were located with a distance of 1 cm from the center of the bifurcation of the PA in order to avoid interference by local flow turbulences in this area. For the comparison of pulmonary and aortic blood flow a fourth analysis plane was positioned in the ascending aorta (AAo) at the level of the lower edge of the left pulmonary artery (see Fig. 1). For each analysis plane, a lumen contour surrounding the lumen was defined manually while the contour was adapted automatically to all time points of the cardiac cycle [15].

### Parameters of pulmonary blood flow

The following parameters were evaluated because they were either regarded as parameters of vascular function or have been used earlier as potential surrogates for elevated pulmonary arterial pressure [5, 6, 9, 10]: 1) minimum (CSA_min_), maximum (CSA_max_), average (CSA_avg_) and change (CSA_change_) of cross-sectional-area of the lumen and the MPA/AAo ratio (CSA_ratio_) as calculated from CSA_min_, 2) time to peak systolic (TTP_sys_) and peak diastolic (TTP_dia_) antegrade flow and time to peak retrograde flow (TTP_ret_) as measured from the beginning of the acquisition (i.e., the QRS), 3) parameters of planar blood-flow (i.e., peak (V_max_) and average (V_avg_) velocities in any direction, peak systolic (Q_max-sys_) and diastolic (Q_max-dia_) flow, mean flow per cardiac cycle (Q_mean_), fraction of retrograde flow (RF), and cardiac output (CO). Additionally, flow was visualized in the MPA plane and analyzed regarding the appearance of vortices (i.e., flow which is mostly rotating on an axis line) and the extent of retrograde flow, which were both associated with increased pulmonary pressure in earlier studies [8–10]. To test correctness of 4D flow CMR measures, conservation of mass was tested by measuring mean flow per cardiac cycle in the MPA (Q_MPA_), LPA (Q_LPA_), RPA (Q_RPA_), and the AAo (Q_AAo_). Provided that 4D flow CMR measurements are accurate, blood flow was expected to be the same during one cardiac cycle at the right and the left outflow tract of the heart: Q_MPA_ = Q_LPA_ + Q_RPA_ = Q_AAo_.

### Statistical analysis

Data are presented as mean (±standard deviations) or median (interquartile range) for continuous, absolute and relative frequencies for categorical variables. Regression analysis was performed to quantify correlations of flow between various blood vessels. Furthermore, patients were categorized according to their age into three groups which were used for comparisons of blood flow: group 1 (20–39), group 2 (40–59), and group 3 (60–80 years of age). Departures from normality were detected with the Shapiro-Wilk statistic. Homogeneity of variance was assessed using Levene’s test. Differences between patient groups were evaluated using Fisher’s exact test and one-way ANOVA with Tukey’s HSD post-hoc test, respectively. Furthermore, trends between groups were tested using linear regression and correlations between age and hemodynamic parameters were assessed after adjustment for cardiovascular risk factors and body-mass-index. All tests were two-sided with 0.05 as the level of statistical significance. Statistical analyses were performed using IBM-SPSS Statistics version 19.0.1.

## Results

### Patient characteristics

Cardiovascular risk factors, patient demographics and results from TTE are presented in Table 1. All participants were caucasian. Hypertension and hypercholesterolemia occurred more often in older subjects (group 3 > 2 > 1) while no differences between groups were observed regarding the remaining risk factors. Only few subjects had diabetes (*n* = 2), prior stroke (*n* = 2), coronary-artery-disease (*n* = 2), and none of the subjects suffered from peripheral vascular disease. In the onsite interview none of the subjects reported a history of relevant lung disease (i.e., asthma, chronic obstructive pulmonary disease, interstitial lung disease, pulmonary embolism, tumor, pleural disease or acute infection of the respiratory tract) at the time of examination. In 54 (42.56 %) patients systolic PA pressure could be quantified from tricuspid regurgitation Doppler. Right ventricular systolic pressure was higher in older patients (group 3 > 2 > 1) but still within the reference range according to the ASE recommendations [13] and none of the patients had signs of PH. Echocardiographic findings were within the reference range in all but two individuals, with one having a mildly abnormal left-ventricular function (EF =45 %) and one having an enlarged left atrium (LA diameter =49 mm).

**Table 1:** Characteristics of study participants

Characteristics of patients	Group 1 (20–39y.) *n* = 43	Group 2 (40–59y.) *n* = 44	Group 3 (60–80y.) *n* = 39	*p*-value
Age, years(±SD)	30.07 (±5.4)	50.43 (±5.5)	68.95 (±5.2)	<0.001*
Female, *n*(%)	19 (44.2)	24 (54.6)	21 (53.9)	0.564
Hypertension, *n*(%)	1 (2.3)	7 (16.3)	13 (33.3)	<0.001*
Hypercholesterolemia, *n*(%)	1 (2.3)	9 (20.5)	11 (28.2)	0.005*
Diabetes, *n*(%)	0 (0.0)	1 (2.3)	1 (2.6)	0.587
Smoker, *n*(%)	11 (25.6)	6 (13.6)	5 (12.8)	0.223
BMI, 1(±SD)	24.02 (±4.0)	26.03 (±4.4)	24.31 (±3.8)	0.051
Prior stroke, *n*(%)	0 (0.0)	2 (4.6)	0 (0.0)	0.151
Coronary heart disease, *n*(%)	0 (0.0)	0 (0.0)	2 (5.1)	0.104
Peripheral arterial disease, *n*(%)	0 (0.0)	0 (0.0)	0 (0.0)	–
Mean systolic BP, mmHg(±SD)	120.63 (±11.1)	126 (±15.7)	133.78 (±19.2)	<0.001*
Mean diastolic BP, mmHg(±SD)	76.53 (±7.0)	81.88 (±8.3)	81.09 (±11.4)	0.014*
Heart rate, bpm(±SD)	67.16 (±7.9)	65.55 (±7.7)	66.1 (±9.1)	0.655
Ejection fraction, %(±SD)	56.6 (±20.7)	57.6 (±19.2)	53.6 (±22.5)	0.669
PI grade I, *n*(%)	3 (7.0)	1 (2.3)	1 (2.6)	0.246
PI grade II- IV, *n*(%)	0 (0.0)	0 (0.0)	0 (0.0)	–
RVSP, mmHg(±SD)	15.82 (±2.4)	18.22 (±4.8)	22.05 (±5.7)	<0.001*
TAPSE, mm(±SD)	22.93 (±4.1)	22.56 (±3.3)	23.06 (±3.7)	0.906
TDI, cm/s(%)	15.46 (±2.9)	15.53 (±1.8)	15.82 (±1.8)	0.592

Demographics, cardiovascular risk factors and results from transthoracic echocardiography of study participants. *SD* standard deviation, *BP* blood pressure, *PI* pulmonary valve insufficiency, *RVSP* right ventricular systolic pressure, *TAPSE* tricuspid annular plane systolic excursion, *TDI* systolic velocity of the right ventricular lateral annulus, *statistically significant

### Conservation of mass during 4D flow quantification

Mean stroke volume of all subjects during one cardiac cycle was 75.5 ± 17.2 ml in the MPA, a total of 74.1 ± 17.5 ml in the LPA (34.0 ± 8.8 ml) plus RPA (40.1 ± 9.6 ml), and 74.3 ± 18.2 ml in the ascending aorta (F(2375) = 0.24, *p* = 0.788). Correlations were high between flow values in the MPA and RPA + LPA (*r* = 0.92; *p* < 0.001), the MPA and the AAo (*r* = 0.88; *p* < 0.001), and the RPA + LPA and the AAo (*r* = 0.91; *p* < 0.001).

### Age related change of flow

**Fig. 2:**
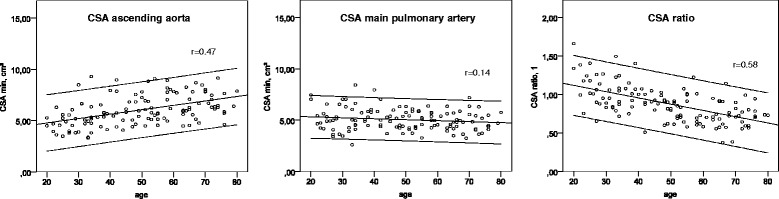
Cross-sectional area of the main pulmonary artery and ascending aorta. Scatter-plots indicating the positive association between diastolic CSA in the ascending aorta and age (*r* = 0.47; *p* < 0.001), the lack of an significant association between the CSA of the main pulmonary artery and age (*r* = 0.14; *p* = 0.11), and the negative association between the CSA ratio and age (*r* = −0.58; *p* < 0.001); the upper and lower line are indicating the 95 % confidence interval of the trend line

The mean cross-sectional area (CSA) of the pulmonary arteries and the ascending aorta is given in Table 2. We observed a trend that the CSA of the MPA was smaller in older subjects, while the CSA of the AAo, LPA and RPA was higher in older participants resulting in a proportionally higher CSA_ratio_ in younger subjects (group 1 > 2 > 3; see Fig. 2). The CSA_change_ decreased with age in the MPA and AAo as a marker of increased stiffness.

**Table 2:** Pulmonary and aortic cross-sectional area

	Group 1 (20–39y.) *n* = 43	Group 2 (40–59y.) *n* = 44	Group 3 (60–80y.) *n* = 39	F(2,375)	*p*-value
CSA_max_, cm^2^(±SD)					
MPA	6.53 ± 1.34	6.11 ± 1.21	5.78 ± 1.17	7.39	0.008*
LPA	3.31 ± 0.65	3.55 ± 1.23	3.69 ± 0.96	3.16	0.078
RPA	3.22 ± 0.62	3.59 ± 1.24	3.79 ± 1.07	6.35	0.013*
AAo	5.97 ± 1.35	7.05 ± 1.51	7.45 ± 1.48	21.41	<0.001*
CSA_min_, cm^2^(±SD)					
MPA	5.19 ± 1.22	5.09 ± 0.96	4.82 ± 0.90	2.99	0.109
LPA	2.71 ± 0.62	2.93 ± 0.98	3.03 ± 0.85	3.08	0.082
RPA	2.46 ± 0.51	2.79 ± 0.99	2.95 ± 0.80	7.86	0.006*
AAo	5.08 ± 1.27	6.28 ± 1.39	6.77 ± 1.45	31.02	<0.001*
CSA_avg_, cm^2^(±SD)					
MPA	5.87 ± 1.27	5.61 ± 1.09	5.29 ± 1.03	5.23	0.024*
LPA	3.03 ± 0.62	3.24 ± 1.12	3.37 ± 0.91	2.59	0.088
RPA	2.88 ± 0.57	3.22 ± 1.13	3.39 ± 0.98	6.19	0.014*
AAo	5.59 ± 1.30	6.78 ± 1.46	7.19 ± 1.47	26.00	<0.001*
CSA_ratio_, 1(±SD)					
MPA/AAo	1.05 ± 0.23	0.83 ± 0.17	0.74 ± 0.19	51.56	<0.001*
CSA_change_, %(±SD)					
MPA	20.78 ± 5.30	16.35 ± 5.04	16.17 ± 5.03	17.06	<0.001*
LPA	18.56 ± 5.59	17.13 ± 3.55	18.29 ± 5.29	0.08	0.773
RPA	23.69 ± 5.52	22.23 ± 5.09	21.64 ± 6.01	2.85	0.094
AAo	15.06 ± 5.85	10.99 ± 4.09	9.26 ± 3.32	32.97	<0.001*

*CSA* cross-sectional-area, *SD* standard deviation, *statistically significant

**Fig. 3:**
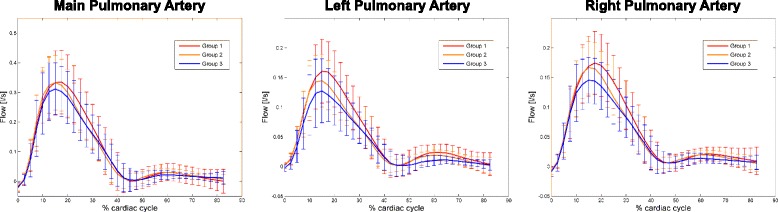
Pulmonary artery flow profiles. The flow profile during the cardiac cycle is given for the main (MPA), the left (LPA), and the right (RPA) pulmonary artery. Groups are represented by different colors (red: group 1 (age 20–39); orange: group 2 (age 40–59); blue: group 3 (age 60–80))

The flow profile in the MPA, LPA, and RPA compromised three phases: 1) systolic antegrade flow, 2) end-systolic and early-diastolic retrograde flow (in all but one patient without any detectable retrograde flow), and 3) diastolic antegrade flow. The distribution of flow in relation to the cardiac cycle is illustrated in Fig. 3 and the position of landmarks (time-to-peak of antegrade systolic, retrograde early-diastolic, and antegrade diastolic flow) is given in Table 3. Peak systolic flow in the MPA occurred later in the cardiac cycle in younger patients (group 1 > 2 + 3) while no difference was observed regarding the time point of peak retrograde flow and peak antegrade diastolic flow.

**Table 3:** Landmarks of the cardiac cycle

	Group 1 (20–39y.) *n* = 43	Group 2 (40–59y.) *n* = 44	Group 3 (60–80y.) *n* = 39	F(2,375)	*p*-value
TTP_sys_, %(±SD)					
MPA	17.25 ± 3.58	15.31 ± 3.02	15.38 ± 3.20	6.70	0.011*
LPA	17.32 ± 6.29	14.65 ± 2.46	15.47 ± 2.50	3.97	0.049*
RPA	18.23 ± 3.52	16.24 ± 2.62	16.56 ± 7.92	2.20	0.140
AAo	15.06 ± 4.59	14.89 ± 5.48	13.46 ± 2.70	2.62	0.108
TTP_ret_, %(±SD)					
MPA	43.52 ± 7.45	43.16 ± 5.91	43.53 ± 4.63	0.00	0.994
LPA	45.26 ± 8.30	44.52 ± 8.29	50.97 ± 13.58	6.37	0.013*
RPA	46.69 ± 10.01	49.02 ± 13.89	49.97 ± 13.65	1.38	0.242
AAo	41.18 ± 9.614	39.51 ± 6.17	40.6 ± 4.33	1.40	0.709
TTP_dia_, %(±SD)					
MPA	59.85 ± 9.296	59.37 ± 6.307	60.53 ± 8.112	0.15	0.701
LPA	61.06 ± 8.991	60.99 ± 7.276	66.76 ± 10.14	8.54	0.004*
RPA	61.72 ± 10.15	62.63 ± 9.908	63.11 ± 10.83	0.38	0.54
AAo	55.71 ± 10.03	57.76 ± 7.792	61.96 ± 9.991	9.26	0.003*

Time-to-peak (TTP) values in % of the cardiac cycle for peak antegrade systolic (TTP_sys_) and diastolic (TTP_dia_), and early diastolic retrograde flow (TTP_ret_). *SD* standard deviation. *statistically significant

Systolic peak flow values (Q_max-sys_) and corresponding velocities (V_max_ and V_avg_) were higher in younger patients (group 1 > 2 > 3) (see Fig. 3 and Table 4) in the left and right pulmonary artery and the AAo. Also, the diastolic peak flow volume was lower in older patients and overall mean flow volume (Q_mean_) and, accordingly, cardiac output as measured in the ascending aorta was higher in younger participants (group 1 > 2 > 3). When comparing hemodynamic parameters between the LPA and the RPA intra-individually, it occurred that peak (V_max_; *p* < 0.001) and average velocities (V_avg_; *p* = 0.001), peak (Q_max-sys_; *p* = 0.003) and mean (Q_mean_; *p* < 0.001) flow were higher in the RPA, while the fraction of retrograde flow was higher in the LPA (*p* < 0.001) (see Table 4).

**Table 4:** Parameters of pulmonary flow

	Group 1 (20–39y.) *n* = 43	Group 2 (40–59y.) *n* = 44	Group 3 (60–80y.) *n* = 39	F(2,375)	*p*-value
V_max_, m/s(±SD)					
MPA	0.88 ± 0.17	0.88 ± 0.16	0.95 ± 0.29	1.86	0.175
LPA	0.85 ± 0.26	0.70 ± 0.16	0.65 ± 0.23	17.74	<0.001*
RPA	0.91 ± 0.18	0.79 ± 0.15	0.78 ± 0.23	9.22	0.003*
AAo	0.71 ± 0.16	0.58 ± 0.15	0.46 ± 0.14	55.34	<0.001*
V_avg_, m/s(±SD)					
MPA	0.23 ± 0.04	0.23 ± 0.04	0.23 ± 0.07	0.68	0.795
LPA	0.23 ± 0.05	0.19 ± 0.03	0.18 ± 0.05	27.40	<0.001*
RPA	0.25 ± 0.05	0.23 ± 0.03	0.21 ± 0.05	21.97	<0.001*
AAo	0.25 ± 0.05	0.23 ± 0.04	0.21 ± 0.05	22.25	<0.001*
Q_max-sys_, mL(±SD)					
MPA	354.2 ± 98.7	344.3 ± 92.6	328.8 ± 93.2	1.46	0.229
LPA	170.4 ± 50.3	150.4 ± 45.6	133.5 ± 56.9	10.73	<0.001*
RPA	186.4 ± 48.5	173.4 ± 44.6	157.3 ± 41.2	8.60	0.004*
AAo	398.5 ± 128.6	343.1 ± 113.6	327.1 ± 85.2	8.39	0.004*
Q_max-dia_, mL(±SD)					
MPA	52.1 ± 18.53	38.6 ± 15.62	29.4 ± 11.17	43.97	<0.001*
LPA	35.0 ± 11.83	22.2 ± 9.04	13.4 ± 8.5	96.55	<0.001*
RPA	28.1 ± 10.78	20.5 ± 9.71	17.1 ± 8.83	25.48	<0.001*
AAo	33.9 ± 14.46	29.5 ± 10.74	18.6 ± 8.07	36.26	<0.001*
Q_mean_, mL/cycle(±SD)					
MPA	79.72 ± 19.41	74.89 ± 17.12	71.65 ± 13.87	4.59	0.034*
LPA	39.01 ± 8.11	33.35 ± 7.76	29.58 ± 8.04	28.04	<0.001*
RPA	43.07 ± 11.05	39.86 ± 8.68	37.04 ± 8.06	8.44	0.004*
AAo	82.21 ± 20.96	73.39 ± 15.64	66.48 ± 13.93	17.13	<0.001*
RF, %(±SD)					
MPA	3.41 ± 2.74	2.09 ± 1.29	2.43 ± 1.76	4.75	0.031*
LPA	2.58 ± 1.65	1.48 ± 1.28	2.03 ± 2.13	2.16	0.144
RPA	1.49 ± 1.20	0.95 ± 0.79	1.66 ± 1.42	0.45	0.504
AAo	1.20 ± 1.12	1.34 ± 1.05	2.25 ± 2.77	7.08	0.009*
CO, L/min(±SD)	5.33 ± 1.56	4.77 ± 0.99	4.39 ± 1.08	11.90	<0.001*

Peak (V_max_) and mean (V_avg_) velocities, peak systolic (Q_max-sys_) and diastolic (Q_max-dia_) antegrade flow volumes, mean flow volume per cardiac cycle (Q_mean_), fraction of retrograde flow (RF), and cardiac output (CO) as measured in the AAo. *SD* standard deviation. *statistically significant

### Age as an independent predictor of hemodynamic change

After adjustment for cardiovascular risk factors (i.e., arterial hypertension and hypercholesterolemia) and body-mass-index, age was found to be an independent predictor of mean flow (Q_mean-MPA_ (*r* = −0.22, *p* = 0.018), Q_mean-LPA_ (*r* = −0.45, *p* < 0.001), Q_mean-RPA_ (*r* = −0.28, *p* = 0.002), Q_mean-AAo_ (*r* = −0.37, *p* < 0.001)), maximum flow (Q_max-LPA_ (*r* = −0.30, *p* = 0.001), Q_max-RPA_ (*r* = −0.24, *p* = 0.008)), fraction of retrograde flow (RF_MPA_ (*r* = −0.22, *p* = 0.017), RF_LPA_ (*r* = −0.26, *p* = 0.004)), cardiac output (*r* = −0.33, *p* < 0.001), mean velocities (V_mean-AAo_ (*r* = −0.47, *p* < 0.001), V_mean-LPA_ (*r* = −0.48, *p* < 0.001), V_mean-RPA_ (*r* = −0.45, *p* < 0.001)), maximum velocities (V_max-AAo_ (*r* = −0.59, *p* < 0.001), V_max-LPA_ (*r* = −0.39, *p* < 0.001), V_max-RPA_ (*r* = −0.32, *p* < 0.001)), minimum CSA (CSA_min-AAo_ (*r* = 0.35, *p* < 0.001)), maximum CSA (CSA_max-MPA_ (*r* = −0.25, *p* = 0.005), CSA_max-AAo_ (*r* = 0.28, *p* = 0.002)), mean CSA (CSA_mean-MPA_ (*r* = −0.22, *p* = 0.017), CSA_mean-AAo_ (*r* = 0.32, *p* < 0.001)), MPA/AAo-CSA ratio (*r* = −0.50, *p* < 0.001) and CSA_change-AAo_ (*r* = −0.41, *p* < 0.001).

### 3D visualization of flow in the main pulmonary artery

**Fig. 4:**
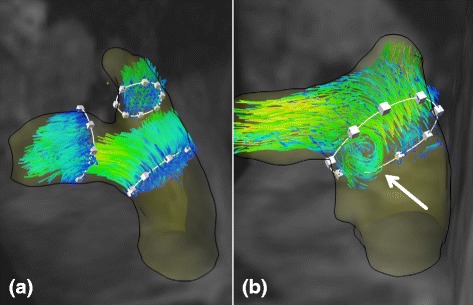
Vortices in the main pulmonary artery. **a** A subject without appearance of vortices in the MPA. **b** A vortex (white arrow) appearing in the MPA during systole

We were able to visualize retrograde blood flow in the main pulmonary artery in all but one participant. In all cases the posterior wall of the MPA was affected by flow reversal. The mean extent of retrograde flow was 0.9–1.4 cm (F(2124) = 0.56; *p* = 0.574). During systole, abnormal vortices (Fig. 4 and Additional file 1: Supplemental video 1) occurred in the MPA in four male subjects with the age of 20, 34, 37, and 60. These subjects had no history of PH and showed no pathological findings in TTE examination.

## Discussion

In this study, we performed 4D flow CMR of the pulmonary arteries in an age-stratified sample of 126 residents of Freiburg, a city in southern Germany with a population of about 230,000. To our knowledge, this is the first population-based study using 4D flow CMR to systematically analyze pulmonary hemodynamics. The purpose of our study was to provide reference values to allow interpretation of findings in future studies using 4D flow CMR, which may both be used as a diagnostic and screening tool if robust prediction of pulmonary hypertension should be possible in the future. 4D flow CMR measures proofed to be highly reliable for the quantification of planar flow in the aorta along with high Intra- and Inter-Observer agreement as shown previously [15]. Furthermore, 4D flow CMR flow measurements have been validated in phantom studies using predefined flow volumes and in comparison with established standard sequences [16]. In our study, the principle of conservation of mass showed excellent agreement when comparing mean flow volumes at the MPA, the combined LPA and RPA, and the AAo along with high correlations. These results indicate that our 4D flow CMR protocol can be robustly used for measuring hemodynamic parameters in the pulmonary arteries.

PA diameter is routinely used to identify PH, as PH is associated with distended proximal pulmonary arteries [17]. However, the definition of PA diameter cut-off values remains problematic. Data from a previous study which examined 51 PH patients (44.8 ± 15 years of age) and 18 not age-matched normotensive controls (53.7 ± 17 years of age) demonstrated that the CSA of the pulmonary arteries is significantly greater in relation to the aortic CSA in PH patients [6]. Our study groups of 40–59 and 60–80 year-old subjects had a similar mean CSA_ratio_ of 0.83 ± 0.17 and 0.74 ± 0.19 when compared with the CSA_ratio_ of 0.87 ± 0.17 in normotensive controls in the study by Boerrigter et al. [6]. They suggested that a dilated PA may be useful for identifying patients with PH. In our study none of the older patients (≥40 years of age) had a CSA_ratio_ ≥1. However, most of the younger participants without evidence for existent PH had a CSA_ratio_ ≥1, because the aortic CSA is usually smaller in young subjects and therefore the ratio is higher. Our data suggests that PA diameter remains relatively stable over time, contrary to aortic diameter which increases with age due to dilatative arteriopathy. Hence, changes of the aortic CSA have to be taken into account when identifying PH patients by an increase in CSA_ratio_. This may be reflected in the results of another study which indicated that the ratio of PA to AAo diameter was no predictor for mortality during a mean follow-up of 3 years [18]. Interestingly, we also detected an age dependent reduction of systolic to diastolic CSA change during the heart cycle, which is a further parameter of vascular stiffness and may be associated with increased mortality in PH patients [17, 19].

Flow in the MPA, LPA, and RPA was triphasic with antegrade flow in systole covering nearly 50 % of the cardiac cycle, a regular short period of flow reversal, and diastolic antegrade flow. Like in other large elastic arteries, the elasticity of the pulmonary arteries contributes to the Windkessel effect which has a reservoir function by saving kinetic energy in systole and releasing it in diastole. The Windkessel effect becomes diminished with age as the pulmonary arteries loose compliance. This results in a reduced elasticity and distensibility in older subjects and explains that time-to-peak systolic flow is shorter compared to younger participants. The lower peak flow volume in older subjects on the other hand can be explained by the age-related reduction of (right-sided) cardiac function. In the absence of significant pulmonary valve insufficiency, retrograde flow was low in the pulmonary arteries and is mainly related to valve closure. The recoil of the pulmonary arteries in diastole results in diastolic antegrade flow which, due to the loss of elasticity, was diminished in older subjects. Moreover, age-related reduction of cardiac function and vascular elasticity explains that older subjects had lower peak and mean velocities, peak systolic and diastolic antegrade flow values, and that mean flow volumes were lower in the MPA, the LPA, and the RPA when compared to younger patients. Barker et al. postulated that peak velocities, peak flow, and stroke volume were lower in PH patients compared to controls. We believe that part of this result may be explained by the effect of ageing, because controls (*n* = 19) were significantly younger than PH patients (*n* = 17) (39 vs. 57 years) in their study [5]. Furthermore, we observed that flow volumes and velocities were higher in the RPA, which can simply be explained by the inherent asymmetry of the lung and lung vascularization with three lobes on the right and two on the left side.

Vortices in the MPA during systole have previously been described to be sensitive and specific for the existence of PH [9]. This information is somewhat underlined by our findings with an absence of vortices of the MPA in 97 % of our subjects. The occurrence of vortices in the MPA in four participants without any pathological findings in TTE may be explained by their individual geometry of the pulmonary bifurcation, which enables the formation of vortices in this area in absence of a pathologic condition. Interestingly, vortices were only detected in male subjects. However, given the low prevalence of vortices in only 3.2 % of all subjects and the post-hoc nature of this finding, it requires an independent validation in a larger sample to substantiate a possible influence of geometry on vortex formation.

Potential limitations of our study were the amount of initial nonresponse to our study invitation. When compared with registry data [20] and data from another German population-based study [21] the subjects under investigation less often had hypertension, hypercholesterolemia, diabetes, were less often smokers, obese, and only few patients suffered from a cardiovascular disease. This was probably due to the recruitment modality which required participants to actively contact the study team and to visit the University Medical Center. Accordingly, particularly healthier or health-conscious residents were probably interested in collaborating in this study. Inclusion of sufficient number of young (20–29 years of age) males is a common problem in cohorts like ours. Accordingly, 14 included subjects were recruited from the personnel of our institution (*n* = 922 male 20–29 years of age) on the basis of first come, first serve to minimize bias. Furthermore, the presence of comorbidity in the older age group may have influenced parameters of pulmonary flow. However, as we are interested in the dependency on age at the population level with comorbidity as a part of the natural aging process and correlations were adjusted for cardiovascular risk factors and body-mass-index, we do not believe that this has an impact on our results. Finally, due to the limited sample size we were not able to address the question of potential sex differences in the different parameters systematically. However, a potential confounding between the effects of age and sex can be excluded due to including roughly the same proportion of female subjects in each age group.

## Conclusions

In conclusion, 4D flow CMR can be used to accurately and comprehensively assess flow in the pulmonary arteries in-vivo. Furthermore, we provided an overview of hemodynamic changes which occur during life in the general population independent from cardiovascular risk factors and body-mass. This effect of ageing should be taken into account when assessing pathologic conditions in the pulmonary arteries in future studies using 4D flow CMR to monitor effects of pulmonary hypertension.

## Abbreviations

AAo, ascending aorta; CSA, cross-sectional-area; LPA, left pulmonary artery; MPA, main pulmonary artery; PH, Pulmonary hypertension; RF, retrograde flow; RPA, right pulmonary artery; TTP, time-to-peak

## Additional file

**Additional file 1:** Supplemental-video I. Occurrence of vortices in systole and retrograde flow in the main pulmonary artery in a subject without cardiovascular risk factors and no pathological findings in TTE. (MP4 2740 kb)
